# Evolving Risk Classifications in AML in a Real-Life Scenario: After Changes upon Changes, Is It More and More Adverse?

**DOI:** 10.3390/cancers15051425

**Published:** 2023-02-23

**Authors:** Clara Aparicio-Pérez, Esther Prados de la Torre, Joaquin Sanchez-Garcia, Carmen Martín-Calvo, Carmen Martínez-Losada, Javier Casaño-Sanchez, Juana Serrano-López, Josefina Serrano

**Affiliations:** 1 Hematology Department, Hospital Universitario Reina Sofía, 14004 Córdoba, Spain; 2 Instituto Maimónides de Investigación Biomédica de Córdoba (IMIBIC), 14004 Córdoba, Spain; 3 Departamento de Ciencias Medico-Quirúrgicas, Universidad de Córdoba, 14004 Córdoba, Spain; 4 Experimental Hematology, Fundación Jiménez-Diaz, UAM, 28040 Madrid, Spain

**Keywords:** acute myeloid leukemia, risk classifications, European Leukemia Net, *TP53* mutations

## Abstract

**Simple Summary:**

Risk classifications in AML models modify definition criteria over time according to the advances in the knowledge of the molecular pathology of the disease. These evolving criteria impact the therapeutic strategy for individual patients. In this study, we aimed to analyze the evolutionary behavior of risk-classification models in a consecutive cohort of unbiased patients.

**Abstract:**

Acute myeloid leukemia (AML) is a heterogeneous disease classified into three risk categories (favorable, intermediate and adverse) with significant differences in outcomes. Definitions of risk categories evolve overtime, incorporating advances in molecular knowledge of AML. In this study, we analyzed the impacts of evolving risk classifications in 130 consecutive AML patients in a single-center real-life experience. Complete cytogenetic and molecular data were collected using conventional qPCR and targeted Next Generation Sequencing (NGS). Five-year OS probabilities were consistent among all classification models (roughly 50–72%, 26–32% and 16–20% for favorable, intermediate and adverse risk groups, respectively). In the same way, the medians of survival months and prediction power were similar in all models. In each update, around 20% of patients were re-classified. The adverse category consistently increased over time (31% in MRC, 34% in ELN2010, 50% in ELN2017), reaching up to 56% in the recent ELN2022. Noteworthily, in multivariate models, only age and the presence of *TP53* mutations remained statistically significant. With updates in risk-classification models, the percentage of patients assigned to the adverse group is increasing, and so will the indications for allogeneic stem cell transplantation.

## 1. Introduction

Acute myeloid leukemia (AML) encompasses a highly heterogeneous hematological malignancy affecting mostly adult patients, with a median age of 68 years [1,2]. AML is caused by accumulating somatically acquired genetic lesions that lead to uncontrolled proliferation of immature hematopoietic progenitor cells. AML diagnostic entities were firstly established only by morphological features, and the Medical Research Council (MRC) group pioneered the integration of conventional cytogenetic findings to define prognostic AML categories [3]. In a pivotal step forward, the European Leukemia Net (ELN) consortium launched, in 2010, specific recommendations for the management of AML patients, while also integrating molecular data from cytogenetic findings, to establish four risk categories [4]. Thus, the presence of mutations in *NPM1* or *CEBPA* was considered to indicate favorable prognosis, and the detection of internal tandem duplication (ITD) in *FLT3* was assigned to the intermediate category, I. Thereafter, advances in molecular methodologies, mainly with Next Generation Sequencing (NGS) panels, allowed us to unravel the complexity of the molecular landscape of AML [5], leading to further refinement of AML risk categories by ELN in 2017 [6]. In this ELN2017 revision, biallelic mutations in *CEBPA* were considered to indicate a favorable prognosis [7], and the impact of ITD-*FLT3* was dissected by the allelic ratio value. Interestingly, new inclusion of adverse prognosis mutations in *RUNX1*, *TP53* and *ASXL1* genes made NGS techniques mandatory to perform correct risk assignment. This ELN2017 risk classification was later widely validated in intensively treated patients in real-life scenarios [8,9]. Recently, a new ELN risk stratification has been launched [10] and includes, for the first time, the so-defined myelodysplasia-related gene mutations [11] in the adverse risk category (*ASXL1*, *BCOR*, *EZH2*, *RUNX1*, *SF3B1*, *SRSF2*, *STAG2*, *U2AF1* and/or *ZRSR2*). Additionally, t(8;16)(p11;p13) was included in the adverse group, and karyotypes with ≥3 trisomies were excluded from this category. In addition, in this recent ELN22 risk classification, the ITD–*FLT3* allelic ratio is no longer considered, but it has always been included in the intermediate-risk group, as was the case in ELN2010. Finally, only mutations in b-ZIP in-frame domain of *CEBPA* gene are assigned to indicate favorable prognosis. Recent validation studies of ELN2022 suggest better indications of clinical outcomes than ELN2017 [12].

All these evolving changes in AML risk classification have two main implications in-real-life scenarios. First is the increasing need for complete cytogenetic, molecular and NGS genetic data, provided by centralized laboratories, which should be available early after diagnosis [13]. Second is that the early risk assignment must be implemented to guide treatment strategies for fit patients. Thus, favorable and intermediate AML patients will receive minimal residual disease (MRD)-guided chemotherapy protocols, whereas for adverse AML patients, there is wide consensus on performing allogeneic hematopoietic stem cell transplantation (HSCT) [10,14]. Patients carrying actionable mutations can additionally receive targeted therapies.

In this work, we aimed to analyze the impacts of evolving risk classifications in the clinical management of 130 consecutive AML patients in a single-center real-life experience. We sought to determine how the application of the current risk stratification model could impact the treatment strategies, especially for fit patients that could benefit for targeted therapies and/or HSCT.

## 2. Materials and Methods

### 2.1. Patients

We included 130 newly adult AML patients diagnosed consecutively in our center between June 2017 and July 2022, studied at diagnosis within the Spanish PETHEMA LAM Diagnostic Platform [13]. Patients with acute promyelocytic or ambiguous lineage leukemias were excluded. AML was diagnosed according to World Health Organization (WHO) 2016 criteria [15], and eligible patients were included regardless of the treatment received. Patients were treated according to PETHEMA current protocols and guides. The prognostic risk was established according to the MRC cytogenetic classification, and ELN2010, ELN2017 and ELN2022 risk stratification [3,4,6,10]. Study protocols were in accordance with the Declaration of Helsinki and approved by the institutional review board. All patients provided written informed consent for inclusion in the clinical and genetic analyses.

### 2.2. Methods

Cytogenetic analysis, including fluorescence in situ hybridization (FISH), was performed in our local laboratory using bone-marrow aspirates obtained at diagnosis. Mutational analysis of *NPM1* and *FLT3* (ITD and TKD) was performed on DNA, using PCR-based methods. Recurrent gene rearrangements, including RUNX1::RUNXT1 and CBFb::MYH11, were analyzed by qRT-PCR and/or FISH. According to ELN recommendations, all patients carrying *NPM1*, *RUNX::RUNXT1 or CBFb::MYH11* were tested for minimal residual disease (MRD) status by qRT-PCR after 1 cycle of intensive treatment.

Testing for recurrent AML mutations, including *ASXL1*, *BCOR*, *EZH2*, *RUNX1*, *SF3B1*, *SRSF2*, *STAG2*, *U2AF1*; and/or *ZRSR2*; and *CEBPA*, *NPM1*, *FLT3* and *TP53*, was performed with the NGS technique, with a limit of detection of 5% variant allele frequency (VAF). A myeloid solution panel (SOPHIA Genetics, Lausanne, Switzerland) and MySeq^TM^Ilumina Platform were uniformly used for NGS analyses. Standardization and validation of genetic analyses were performed in the cooperative Network of PETHEMA AML diagnosis platform as previously described [13]. Further data are detailed in Supplemental Material.

### 2.3. Statistical Analyses

A chi-square test was used to assess associations between categorical variables, and the Wilcoxon rank-sum or Kruskal–Wallis test for continuous variables. Analyses of treatment outcomes used commonly accepted definitions of complete remission (CR) and overall survival (OS) [16]. Survival analyses were performed using the Kaplan–Meier method and compared groups by the log-rank test. The Cox proportional-hazards model was used to evaluate the risk of death among groups. We used ROC curves to compare the ELN2022 and ELN2017 outcome prediction by OS and to achieve CR. A *p*-value < 0.05 was considered as statistically significant. Statistical analyses were performed using SPSS version 22 (IBM, Armonk, NY, USA) and R version 4.2.2 (R Foundation for Statistical Computing, Vienna, Austria) software programs.

## 3. Results

### 3.1. Baseline Patient Characteristics

This study included a total of 130 adult patients consecutively diagnosed newly with AML at our institution. They were studied in diagnostic Spanish Plataform PETHEMA and treated using current protocols. The main characteristics of global cohort and additional baseline dates across ELN2022 risk groups are summarized in Table 1. ELN2022 risk was considered favorable in 15.4% of patients (*n* = 20/130), intermediate in 28.5% (*n* = 37/130), and adverse in 56% (*n* = 73/130).

The median age for the global cohort was 65 (range 18–94) years. The ELN2022 adverse group were older (median age 69 years (range: 30–94)) than intermediate or favorable patients (*p* < 0.001). In the same way, this adverse group constitutes a higher percentage of patients >60 (79.5%) and has male predominance (65.8%), both reaching statistical significance (*p* < 0.001 and *p* = 0.009, respectively). Patients from the adverse-risk category had a lower leukocyte count (*p* = 0.023) than patients in the favorable and intermediate categories.

Most patients were diagnosed as de novo AML: 74.6% (*n* = 97/130) in the global series. However, secondary AML was more frequent in the adverse group (34.2%; *n* = 25/130) compared to intermediate and favorable groups (13.5% and 15%, respectively) (*p* = 0.012). Ninety percent of patients (*n* = 118) received treatment: intensive chemotherapy (IC) in 67% (*n* = 87) and low intensity-HMA based therapy in 24% (*n* = 31) according to clinical criteria. Sixty six percent (*n* = 58/87) of patients who were intensively treated reached CR/CRi after induction therapy. There was a lower rate in the adverse-risk group than intermediate and favorable ones (49%, 76% and 94%, respectively) (*p* = 0.003). Fifty-three patients underwent hematopoietic stem cell transplantation (HSCT). Similar percentages of patients belonged to intermediate and adverse groups.

### 3.2. Mutation Characteristics and Distribution

Most patients (98.4%) had at least one mutation or genetic alteration at diagnosis detected by NGS, PCR and/or cytogenetics analysis. The median number of mutations detected by NGS was two (range 0–6). The most prevalent individual mutations in the global cohort were *IDH1/2* (26.2%), *FLT3*-ITD/TKD (23.8%) and *TP53* (19.2%), followed by *NPM1*, *TET2* and *RUNX1* (17.7%, 17.7% and 15.4%, respectively). Patients from the ELN2022 adverse-risk AML group had more mutations per patient (median 3 (range 1–6)) compared to other groups, though the differences between the groups did not reach statistical significance (*p* = 0.145).

**Figure 1:**
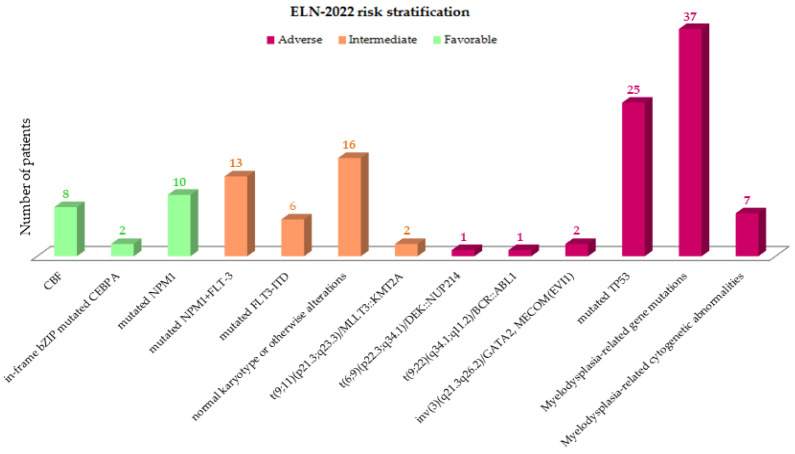
Genetic entities defining in the ELN2022 risk stratification.

Genetic entities defined by the International Consensus Classification (ICC) 2022 groups [17] within the ELN2022 risk classes are represented in Figure 1. The most frequent was AML with myelodysplasia (MDS)-related gene mutations (*n* = 37, 28.5%) and AML with mutated *TP53* (*n* = 25; 19.2%). AML with mutated *NPM1* was the most frequent in the favorable group (*n* = 10; 7.7%). Within the intermediate subgroup, AML, *NPM1* and *FTT3-ITD* co-mutations were the more representative ones (*n* = 13; 10%).

**Figure 2:**
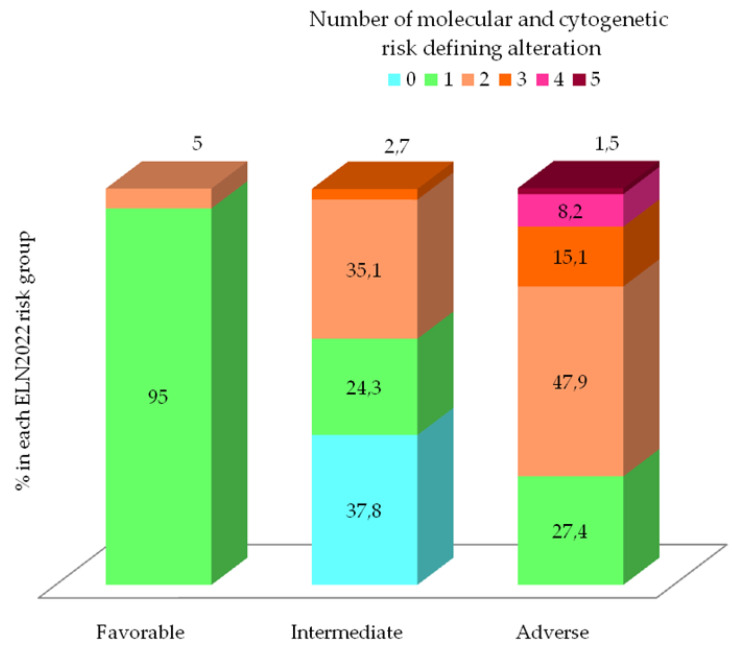
Number of molecular and cytogenetic risk defining alteration according to ELN2022 and distribution acrooss ELN2022 prognostic risk group.

The distribution of the number of risk-defining genetic event according to ELN2022 classification is represented in Figure 2. In the favorable group, 95% of patients (*n* = 17) had only one cytogenetic or molecular abnormality corresponding to *CBF* rearrangement or *NPM1* mutation (median 1, range 1–2). However, in the intermediate group, the median number of genetic events was one (range 0–3), although remarkably, no risk-defining abnormalities were found in 14 patients (37.8%); other recurrent AML mutations not included in the ELN2022 stratification (*IDH1/2*, *TET2*, *DNMT3A* or No-bZIP *CEBPA*) were present in 78% of these patients (*n* = 11/14). In contrast, 72.6% of patients in adverse group presented two or more risk-defining genetic events, showing a statistically difference compared to favorable and intermediate groups (median number of mutations per patient 2 (range 1–5) (*p* < 0.01).

Grouped by functional mutation groups, the most frequent were those related to DNA methylation (46.1%) and signaling/kinase pathways (36.1%). We did not find differences in the distribution of mutations related to DNA methylation and transcription factors by age, but in younger patients there was a higher percentage of those related to signaling pathways, nucleophosmin and transcription factors, which are associated with better prognosis. In contrast, in older patients, unfavorable functional groups (such as chromatin modifiers, spliceosome and *TP53*) are more frequent, reaching statistical significance (Table 2).

Patients older than 60 years presented higher percentages of unfavorable *ASXL1* (21%), *RUNX1* (19.7%) and *TP53* (24.7%) mutations with respect to younger ones (4.1%, 8.1% and 10.2%). These differences were statistically significant in *ASXL1* and *TP53* (*p* = 0.010 and *p* = 0.049, respectively). Some unfavorable MDS-related gene mutations (such as *UA2F1* and *ZRSR2*) did not occur in younger patients in our series. In contrast, *NPM1* (28.6%) and *FLT3*-ITD (32.6%) mutations were significantly more frequent in younger patients (*p* = 0.008 and 0.002, respectively) than in >60-year-old ones (11.1%). Regarding IDH1/2, no major differences were observed between age groups (24.5 vs. 27.1%); see Figure S1.

### 3.3. Evolving Prognostic Risk Classifications

**Figure 3:**
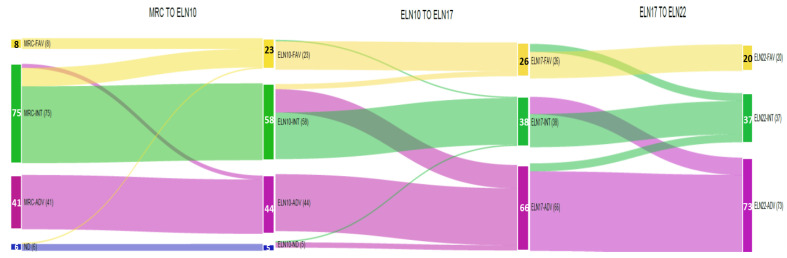
Sankey diagram showing evolving re-classifications from MRC to ELN2022 stratification. Orange: favorable risk; green: intermediate risk; violet: adverse risk; blue: no classified.

According to successive prognostic classifications (MRC cytogenetics, ELN2010, ELN2017 and ELN2022), a higher percentage of patients were considered part of the unfavorable group: 31.5%, 33.8%, 50.8% and 56.1%, respectively. In contrast, the intermediate-risk group decreased over time: 57.6%, 46.5%, 29.2% and 28.5% by the more recent ELN2022 risk categorization. Finally, patients classified as part of the favorable risk group (15.4%) also slightly decreased in proportion compared to the ELN2017 (20%) and ELN2010 (23%) (Figure 3 and Table S1).

From the initial MRC cytogenetic classification, a significant number of patients of the intermediate group (*n* = 14) were allocated to the favorable-risk group by the ELN2010 due to the presence of *NPM1* and *CEBPA* mutations. Thereafter, a significant proportion of patients (*n* = 18) moved from the ELN2010 intermediate group to the ELN2017 adverse group due to the presence of mutations in *TP53*, *ASXL1* or *RUNX1* genes. Finally, the ELN2022 adverse group was further filled-up with the transition of 13 additional patients moving from the ELN2017 intermediate-risk group, due to the presence of mutations in any of the MDS-related genes. However, the ELN2022 intermediate group was still maintained at the same percentage as the ELN2017 one due to the incorporation of all ITD-*FLT3* (*n* = 10), regardless of allelic ratio and *NPM1* co-mutation coming from favorable and adverse ELN2017 risk groups. Additionally, two patients with *biallelic CEBPA* mutation moved from the favorable ELN2017 category to the intermediate ELN2022 one due the lack of in-frame b-ZIP *CEBPA* gene mutations.

Based on the ELN2022, 19.2% (*n* = 25) of patients were re-stratified to another prognostic category with respect to ELN2017, resulting in re-stratification of 23% of favorable ELN2017 (*n* = 6/26), 34.2% of intermediate ELN2017 (*n* = 13/38) and 9.1% of adverse ELN2017 (*n* = 6/66) prognostic groups (Figure S2). Formerly, 21.5% (*n* = 28) of patients were reassigned from ELN2010 to ELN2017, and 13.8% (*n* = 18) were when changing from MRC to ELN2010. Regarding competing ELN risk-defining mutations, two patients with an *NPM1* mutation and MDS-related gene mutations without high-risk cytogenetic alterations were considered to have a favorable prognostic risk. Other cases of “controversial” mutational states for ELN2022 risk categorization are summarized in Table S2. Regarding MRD assessment in *NPM1* and CBF-AML cases, three patients showed positivity by qRT-PCR after two cycles of treatment and were reassigned to undergo allogeneic HSCT.

### 3.4. Overall Survival According to Evolving AML Risk Categories

**Figure 4:**
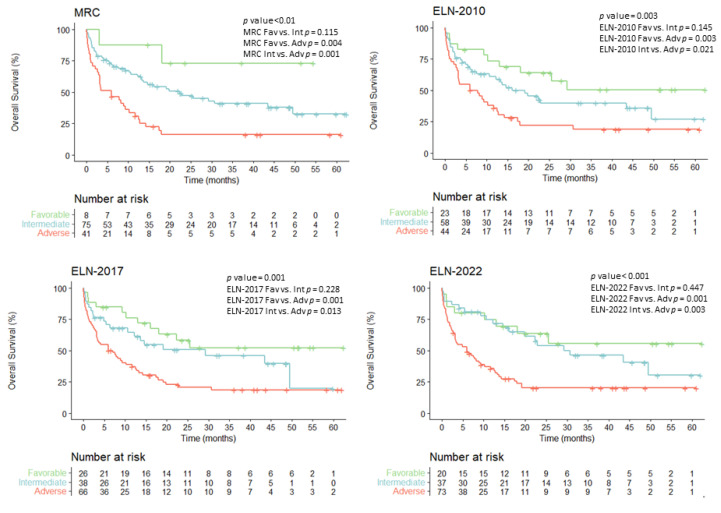
Overall survival probability for the entire cohort (*n* = 130) according to the evolving risk classifications.

The median follow-up of our global cohort (*n* = 130) was 32 months (range: 1–62). The overall survival (OS) mean was 25.4 months (95% confidence interval (CI) 20.7–30.2), and median survival was 13.06 months (95% CI 8.2–17.9) (Figure S3). Overall survival (OS) analyses of the entire cohort (*n* = 130) showed statistically significant differences for all the different risk classifications: MRC, ELN2010, ELN2017 and ELN2022 (*p* < 0.001, *p* = 0.003, *p* = 0.001 and *p* < 0.001, respectively) with OS probabilities of roughly 50–72%, 26–32% and 16–20% for favorable, intermediate and adverse risk groups, respectively (Figure 4).

Detailed data about overall survival at 2 and 5 years for the global series and according to MRC, ELN2010, 2017 and 2022 stratifications are summarized in Table S3.

In all classification systems, the favorable group showed a reproducible OS mean of 37–40 months A more heterogenous median OS was observed in the intermediate groups, ranging from 19.0 months for ELN2010 to 30.6 months for ELN2022. In sharp contrast, adverse group showed a consistent OS survival median of 6 months. Statistical differences were observed when comparing the adverse group with favorable and intermediate groups in all classification models, but differences did not reach statistical differences when comparing favorable and intermediate-risk groups.

### 3.5. Overall Survival for Intensively Treated Patients According to Evolving AML Risk Categories

**Figure 5:**
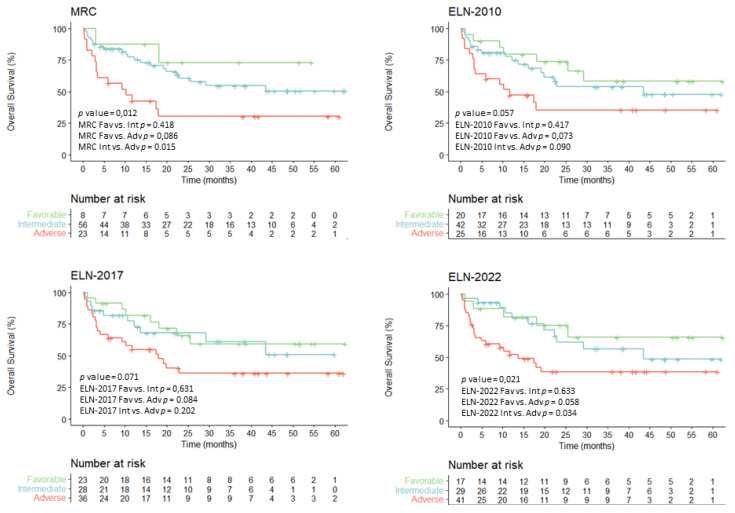
Overall survival probability for intensively treated patients (*n* = 87) according to the evolving risk classifications.

All ELN AML risk classifications have been developed based on data from intensively treated patients. In this sense, survival analysis was performed by selecting our patients who received intensive chemotherapy (*n* = 87). They showed OS at 5 years of 65.7%, 48.5% and 38% for favorable, intermediate, and adverse ELN2022 risk groups, respectively, maintaining statistical significance (*p* = 0.021) (Table S4). These data also represent an improvement compared to the ELN2017 categorization (favorable 59.4%, intermediate 50.1% and adverse 36.1%, *p* = 0.062) (Figure 5).

However, patients who underwent hematopoietic stem cell transplantation (HSCT) (*n* = 53) showed similar OS at 5 years in intermediate and adverse ELN2022 risk groups (56.4% and 52.7%, respectively; *p* = 0.222), suggesting that HSCT could abrogate the dismal prognosis of the unfavorable group (Figure S4). Only six patients within the favorable ELN2022 risk group received HSCT (due to molecular or hematological leukemia relapse) and had an improved overall survival rate (83%). According to the ELN2017, favorable and intermediate prognostic groups had comparable survival (OS at 5 years: 66.7% in favorable and 64.7% in intermediate) after HSCT.

We did not find statistical differences in predicting OS when comparing ELN2022 and 2017 for the global series (*p* = 0.781) (Figure S5) (ROC for OS—ELN2022 AUC: 0.659 (0.56–0.757); ELN2017 AUC: 0.668 (0.57–0.765)), and there were similar results in patients receiving intensive chemotherapy (ROC for OS in intensive treated patients—ELN2022: AUC 0.619 (0.5–0.737); ELN2017 AUC: 0.615 (0.495–0.73).

In our series, patients receiving low-intensity HMA-based therapies had a median OS of 7 months; CI 95% 4.9–9.1. Risk assessment models showed no statistical differences for non-intensively treated patients (*n* = 31) (Figure S6). Thus, ELN2022 classification showed median overall survival of 9 months (CI 95% 7.2–16.8), 14.6 months (CI 95% 0–34.4) and 6 months (CI 95% 3.8–8.1) for favorable, intermediate and adverse group, respectively (*p =* 0.06). Similar median OS data were obtained using ELN2017 and ELN2021: 9 months, 6 months and 5.9 months for favorable, intermediate and adverse risk, respectively (*p* = NS).

### 3.6. Univariate and Multivariate Analyses

In univariate analyses for OS, clinical variables with statistical significance were age and all successive risk classifications. No differences in estimated OS rate were detected according to sex, clinical subtype (de novo vs. secondary AML) or WBC in intensively treated patients. We did not observe differences within favorable risk ELN2022 sub-groups (*p* = 0.128). The estimated 5-year OS rate was between 47 and 73%. Similarly, comparison between the intermediate-risk ELN2022 entities did not show significant differences in outcome among subsets (*p* = 0.601). However, we observed clearly different OS rates (*p* = 0.007) in adverse genetic subgroups. There was significantly worse survival in patients carrying *TP53* mutations (median OS: 2.7 months, CI 95% 0.1–5.5).

The multivariate analysis (Table 3) confirmed the independent prognostic impact on OS of age (>60 years HR 2.95). With respect to ELN2022 risk stratification, although the HR of death in the univariate analysis increased from 1.36 to 3.24 in the intermediate- and adverse-risk groups (*p* = 0.002), these data did not reach statistical significance in multivariate analysis (HR 1.24 to 1.7; *p* = 0.201) (Figure S7).

**Figure 6:**
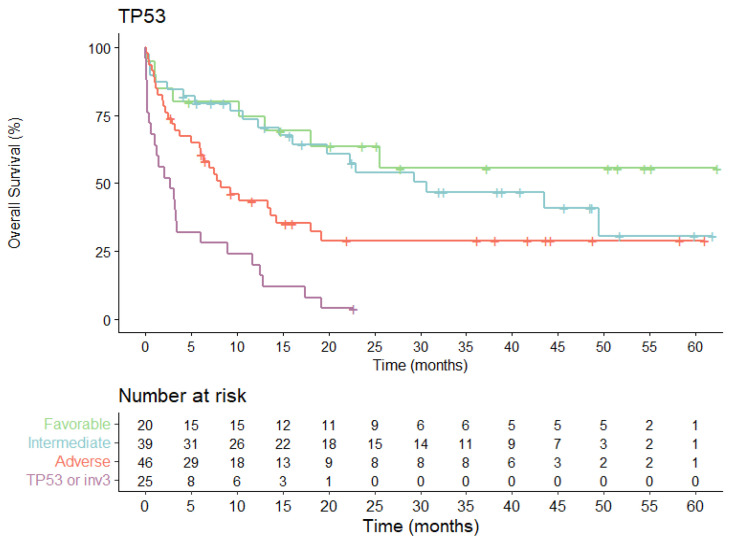
Overall survival probability for the entire cohort (*n* = 130) according to the ELN2022 high-risk group defined by the presence of *TP53* mutation.

*TP53* mutation indicated extremely poor prognosis in multivariate analysis (HR 3.17, 95% CI: 1.52–6.6; *p* = 0.001), being the only molecular alteration and classification group that maintained an independent prognostic impact in this analysis (Figure 6).

## 4. Discussion

Evolving risk classifications designed for patients diagnosed with AML have classically defined three categories (favorable, intermediate, and adverse) with significant different outcomes [3,4,6,10]. In our series of unbiased consecutive patients, 5-year OS probabilities are consistent though all classification models (roughly 50–72%, 26–32% and 16–20% for favorable, intermediate and adverse risk groups, respectively). In the same way, the medians of survival months and prediction power were similar for all models. The main difference was the proportion of patients assigned to each prognosis group in the evolving models. Thus, the adverse category consistently increased over time (31% in MRC, 34% in ELN2010, 50% in ELN 2017), reaching up to 56% in the recent ELN2022. Conversely, the percentage of intermediate-risk patients progressively decreased (58% in MRC, 46.5% in ELN2010, 29% in ELN 2017 and 28% in ELN2022). With the evolving risk classifications, 14–21% of patients were re-classified, and thus, the treatment strategy also must change. In particular, the indication of HSCT at first complete remission for patients moved from favorable to intermediate-adverse groups. Noteworthily, these classification models defined statistically different categories for outcomes in univariate analyses, but in multivariate models, only age and presence *TP53* mutations remained significant.

In AML, it is broadly assumed that, mostly in fit patients, genetic lesions account for about two-thirds of OS variation, and the other third were contributed by demographic, clinical and treatment variables [18]. Thus, these different evolving risk categories have been validated for intensively treated patients and only consider the presence of genetic lesions. Since first incorporation of cytogenetic abnormalities in the MRC risk classification, ELN proposals have been progressively adding more and more genetic lesions (up to 32 in the last ELN2022 update). It has become mandatory to accurately assignment of risk. This assumption implies that diagnostic laboratories must provide cytogenetic and genetic data of AML at diagnosis in a timely fashion, to allow clinicians to make early therapeutical strategies. From a practical point of view, NGS is still non-affordable for many institutions due to a still elevated cost, the needing of batching samples and time-consuming running and reporting results requiring expertizing [19]. Therefore, referring samples to centralized harmonized specialized laboratories is currently the most common approach for getting NGS results early during the cycle of treatment [13].

The favorable risk group encompassed a stable percentage of all AML patients, approximately 20% of cases through all ELN updates. While CBFB::MYH11 and RUNX1::RUNX1T1 rearrangements have been always maintained as a favorable group, prognosis of *CEBPA* gene mutations has been redefined over time from single mutation (sm) to double mutation (dm), and eventually went back to sm but in-frame, affecting the b-ZIP domain [7,20,21]. Likewise, prognosis of *NPM1* mutations has also been redefined over time considering the co-existence and ratio of ITD in *FLT3* genes. Despite being considered of favorable risk, this group showed a 5-year OS of just 50–55%, pointing out a clear field of improvement. The presence of co-mutations (*C-KIT*, *DNMT3A*, *GATA1*, etc.) conferring worse outcomes have not been considered in any risk classifications. Inadequate MRD clearance is a surrogate dynamic marker of worse prognosis [22].

The intermediate group constituted a heterogenous population with a median 5-year OS ranging from 19.0 months in ELN2010 to 30.6 months in ELN2022. The optimal post-remission therapy in intermediate-risk AML is still a matter of debate, and the potential beneficial role of allogeneic HSCT has not been uniformly supported [14,23,24]. Evolving classifications reduce progressively the percentage of patients assigned to this category, being 28% for the current ELN2022. The largest group of AML patients carried ITD-*FLT3* mutations regardless of the allelic ratio [25] or *NPM1* mutation (as it was considered in ELN2010). Thus, uncertainties are currently open about the current indication of HSCT for patients, with ITD-*FLT3* receiving midostaurin and adequate *NPM1* MRD clearance [26,27].

As previously mentioned, the percentage of patients classified as adverse group reached up 56% according to the recent ELN2022 criteria, suggesting that, in more than half of fit patient candidates for intensive treatment, allogeneic HSCT will be indicated [28]. Patients are assigned to this adverse category mostly due to MDS-related-gene mutations, high-risk cytogenetic findings and/or *TP53* mutations. In the pivotal work of Lindsley et al. [11], the presence of *SRSF2*, *SF3B1*, *U2AF1*, *ZRSR2*, *ASXL1*, *EZH2*, *BCOR* or *STAG2* was >95% specific for the diagnosis of s-AML. Importantly, in the majority of patients with s-AML achieving morphological remission, these driver mutations are still detectable, supporting the hypothesis that these patients may have had an unrecognized period of antecedent myelodysplasia. With this premise, the presence of mutations in these genes has been recently incorporated to adverse risk in ELN2022 recommendations, notably increasing the percentage of patients in this category. Notwithstanding the fact that mutations in these genes can be detected in other well-defined genetic categories, the benefit of specific s-AML approved treatments and the potential curative role of allogeneic HSCT still remain unanswered questions. AML with *TP53* mutations have been recently recognized as a different diagnostic entity [17], and its very adverse prognosis, incorporated in EL2017 risk recommendations, has been observed throughout all clinical series [29,30,31,32,33].

As limitations of our study, the number of consecutive patients diagnosed in a single institution surely was not large enough to draw firm conclusions, but our data reflect in a real-life setting the risk-classification models. Future directions in this scenario will include the validation of the most recent classification, ELN2022: (i) confirmation of the prognostic integration of bZIP *CEBPA* mutations; (ii) the long-term follow-up of patients treated with *FLT3* inhibitors which could overcome prognostic value; (iii) confirmation of adverse outcomes in patients with MDS-related genes mutations; and finally, the possible definition of very-adverse subgroup.

## 5. Conclusions

Evolving risk-classification proposals clearly identify three categories of patients with different outcomes, especially among those patients who are intensively treated. The percentage of patients classified in the adverse group is increasing, being up to 56% in the recent ELN2022, and conversely, the percentage of intermediate-risk patients has progressively decreased. With the evolving risk classifications, 14–21% of patients have been re-classified, and thus, their treatment strategy also must change. Noteworthily, these classification models defined statistically different categories for outcomes in univariate analyses, but in multivariate models, only age and the presence of *TP53* mutations remained clearly significant.

## Figures and Tables

**Table 1:** Patient baseline, treatment, and prognostic characteristics of the entire cohort according to the ELN2022 risk stratification.

*n* (%)	Cohort 130 (100)	ELN2022 Favorable 20 (15.4)	ELN2022 Intermediate 37 (28.5)	ELN2022 Adverse 73 (56.1)	*p* ^†^
Age, years median (range)	65 (18–94)	50 (25–89)	55 (18–89)	69 (30–94)	<0.01
Age ≥ 60 years	82 (63.1)	8 (40)	16 (43.2)	58 (79.5)	<0.01
Sex, male *n* (%)	71 (54.6)	10 (50)	13 (35.1)	48 (65.8)	0.009
WBC × 10^9^/L, median (range)	10.4 (0.5–590)	30 (1.2–158)	22.4 (0.9–279)	7.2 (0.5–590)	0.02
Clinical subtypes *n* (%)					
De-novo	97 (74.6)	17 (85.0)	32 (86.5)	48 (65.8)	0.01
s-AML	21 (16.2)	0 (-)	2 (5.4)	19 (26.0)	-
t-AML	12 (9.2)	3 (15)	3 (8.1)	6 (8.2)	-
NPM1 *n*(%)	23 (17.7)	10 (50)	13 (35.1)	0 (0)	<0.01
FLT3-ITD *n* (%)	23 (17.7)	1 (5)	16 (43.2)	6 (8.2)	<0.01
Received treatment *n* (%)					
Intensive chemotherapy (IC)	87 (66.9)	17 (85)	29 (78.4)	41 (56.2)	0.01
HMA-based/low intensity	31 (23.9)	3 (15)	5 (13.5)	23 (31.5)	-
Supportive care	12 (9.2)	0 (0)	3 (8.1)	9 (12.3)	-
HSCT *	53 (61)	6 (35)	20 (69)	27 (66)	0.13
Complete Remission (CR/CRi) *	58 (66.6)	16 (94.2)	22 (75.8)	20 (48.8)	<0.01
Relapse rate *	23 (26.4)	4 (23.5)	10 (34.5)	9 (33.3)	0.78
Exitus rate	80 (61.5)	8 (40)	19 (51.4)	53 (72.6)	<0.01

ELN2022: European Leukemia Net 2022; WBC: white blood count; NPM1: nucleophosmine 1; FLT3-ITD: fms-like-tirosin kinase 3 internal tandem duplication; s-AML: secondary AML; t-AML: therapy-related AML; HMA: hypomethylating agents; HSCT: hematopoietic stem cell transplantation; * percentage calculated relative to the intensive treatment patient. ^†^ *p* value for three groups comparisons.

**Table 2:** Frequencies functional mutations groups in global cohort and grouped by age.

Functional Mutations Group *n* (%)	Mutations	Cohort (*n* = 130)	<60 Years (*n* = 49)	≥60 Years (*n* = 81)	*p*
Signaling pathways	*FLT3*, *KRAS*, *NRAS*, *KIT*, *PTPN*	47 (36.1)	23 (46.9)	24 (29.6)	0.03
Epigenetic modification					
DNA methylation	*DNMT3A*, *IDH1/2*, *TET2*	60 (46.1)	22 (44.8)	38 (46.9)	0.92
Chromatin modifiers	*ASXL1*, *EZH2 y MLL/KMT2A*	19 (14.6)	3 (6.1)	16 (19.7)	0.04
Nucleophosmin	*NPM1*	23 (17.7)	14 (28.5)	9 (11.1)	<0.01
Transcription factors	*CEBPA*, *RUNX1 y GATA2*	24 (18.5)	7 (14.3)	17 (21)	0.37
Tumor Suppressors	*TP53*	25 (19.2)	5 (10.2)	20 (24.6)	0.05
Spliceosome complex	*SRSF2*, *U2AF1*, *SF3B1 y ZRSR2*	32 (24.6)	3 (6.1)	29 (35.8)	<0.01
Fusiontranscription factors	*RUNX1/RUNX1T*, *MYH11/CBF*	8 (6.1)	6 (12.3)	2 (2.5)	0.02

**Table 3:** Univariate and multivariate analysis for overall survival.

Variable		Univariate		Multivariate	
		OR(95% CI)	*p*	HR(95% CI)	*p*
Age (>60 years)		3.97 (2.31–6.86)	<0.001	2.95 (1.65–5.3)	<0.01
TP53 mutation		3.71 (2.56–8.8)	<0.001	3.17 (1.52–6.6)	0.001
ELN-2022 risk stratification	Favorable	reference	-	reference	-
	Intermediate	1.36 (0.59–3.1)	0.47	1.24 (0.54–2.86)	0.613
	Adverse	3.24 (1.53–6.86)	0.002	1.7 (0.75–3.82)	0.201

## Data Availability

The data presented in this study is available within the article and Supplementary Materials.

## Supplementary Materials

The following supporting information can be downloaded at: https://www.mdpi.com/article/10.3390/cancers15051425/s1. DNA extraction; Genes regions analyzed by the Myeloid Solution panel; Figure S1: Frequency of NGS mutations in a global cohort grouped by age; Figure S2: Bar plot representing the percentage of patients re-stratified according to ELN2022 in ELN2017 risk groups (A) and overall ELN2022 risk groups (B); Figure S3. Overall survival for the entire cohort (*n* = 130); Figure S4. Overall survival according to risk categories and classifications for patients receiving HSCT (*n* = 53); Figure S5: ROC curves for OS in a global cohort by ELN2022 and ELN2017; Figure S6: Overall survival according to risk categories and classifications for patients receiving low-intensity treatment (*n* = 31); Figure S7. Overall survival HRs obtained by multivariate analyses; Table S1: Patient distribution of the entire cohort and according to ELN 2022 risk stratification with previous risk classifications; Table S2: Controversial mutational states according to ELN2010, ELN2017 and ELN2022 risk stratification; Table S3: Overall survival rates after 2 and 5 years, with estimated mean and median survival of the global cohort. detailed for each MRC cytogenetic, ELN2010, ELN2017 and ELN2022 risk group; Table S4: Overall survival rates after 2 years and at 5 years and estimated mean and median of the intensively treated patients. detailed for each MRC cytogenetic, ELN2010, ELN 2017 and ELN 2022 risk group.
