# Supplementary material for: Evolving Risk Classifications in AML in a Real-Life Scenario: After Changes upon Changes, Is It More and More Adverse?
Source: Cancers (Basel). 2023 Feb 23;15(5):1425. doi: 10.3390/cancers15051425 (PMC10001051; doi:10.3390/cancers15051425)
Supplement: Supplementary file 1 [file cancers-15-01425-s001.zip › cancers-2205455-supplementary.pdf]

Supplementary Materials: Evolving risk-classifications in AML in a real-life scenario: After changes upon changes, is it more and more adverse?

Clara Aparicio-Pérez, Esther Prados de la Torre, Joaquin Sanchez-Garcia, Carmen Martín-Calvo, Carmen Martinez-Losada, Javier Casaño-Sanchez, Juana Serrano-López and Josefina Serrano

DNA Extraction

Nucleic acid isolation DNA from white blood cells was obtained in each center following previously established DNA isolation protocols. DNA quantification was assessed with Nanodrop (Thermo Fisher Scientific, Waltham, MA USA) and Qubit fluorometer (Thermo Fisher Scientific). DNA integrity was assessed with Bioanalyzer (Agilent Technologies, Santa Clara, CA, USA) or Tape Station 4100 (Agilent Technologies).

Genes Regions Analyzed by Myeloid Solution Panel

ABL1 (4–9), ASXL1 (10,12,13), BRAF (15), CALR (9), CBL (8,9), CEBPA (all), CSF3R (all), DNMT3A (all), ETV6 (all), EZH2 (all), FLT3 (13–15,20), HRAS (2,3), IDH1 (4), IDH2 (4), JAK2 (all), KIT (2,8–11,13,17,18), KRAS (2,3), MPL (10), NPM1 (10,11), NRAS (2,3), PTPN11 (3,7–13), RUNX1 (all), SETBP1 (4), SF3B1 (10–16), SRSF2 (1), TET2 (all), TP53 (all), U2AF1 (2,6), WT1 (6–10), ZRSR2 (all).

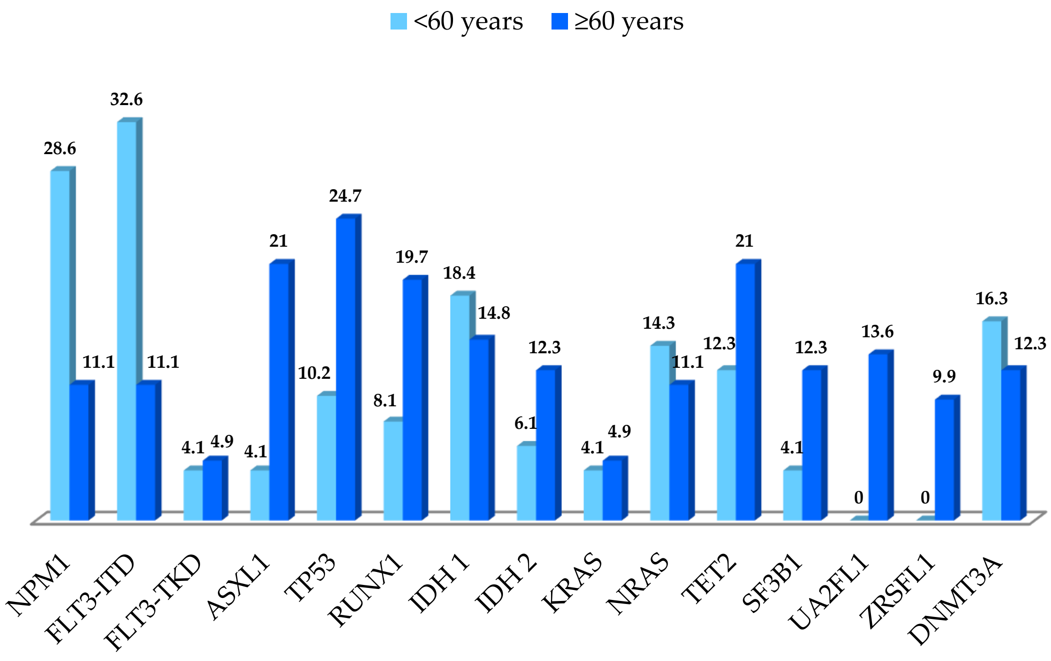

Figure S1. Frequency of NGS mutations in global cohort grouped by age.

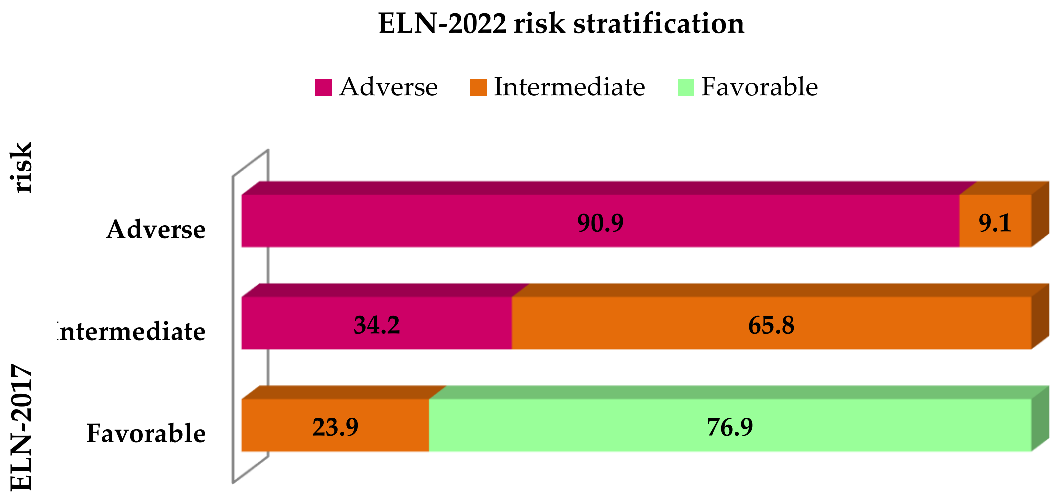

Figure S2. Bar plot representing the percentage of patients re-stratified according to ELN2022 in ELN2017risk groups.

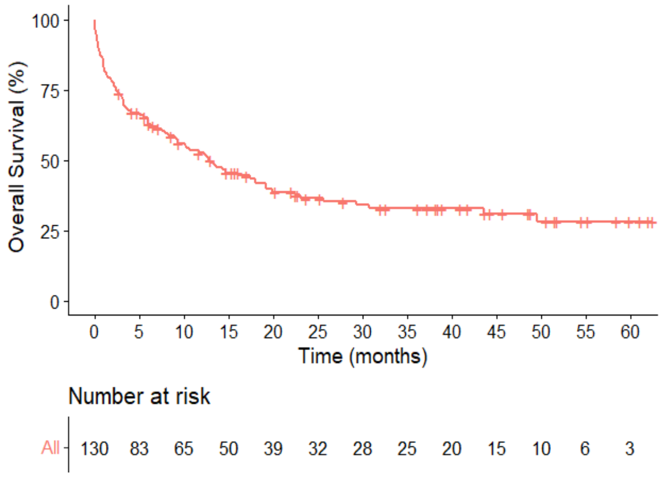

Figure S3. Overall Survival for the entire cohort (n = 130).

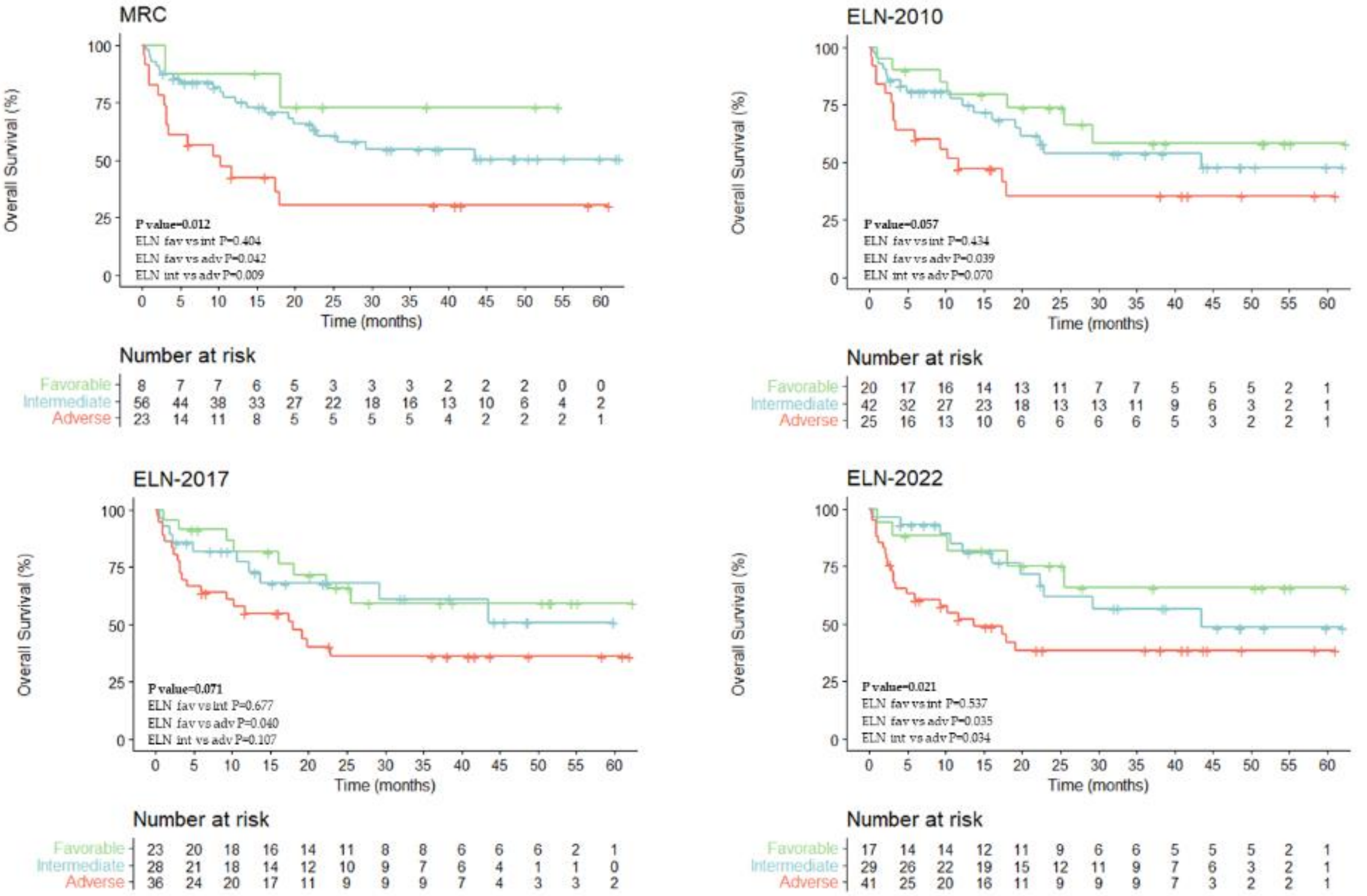

Figure S4. Overall Survival according to risk categories and classifications for the intensively treated patients (n = 87).

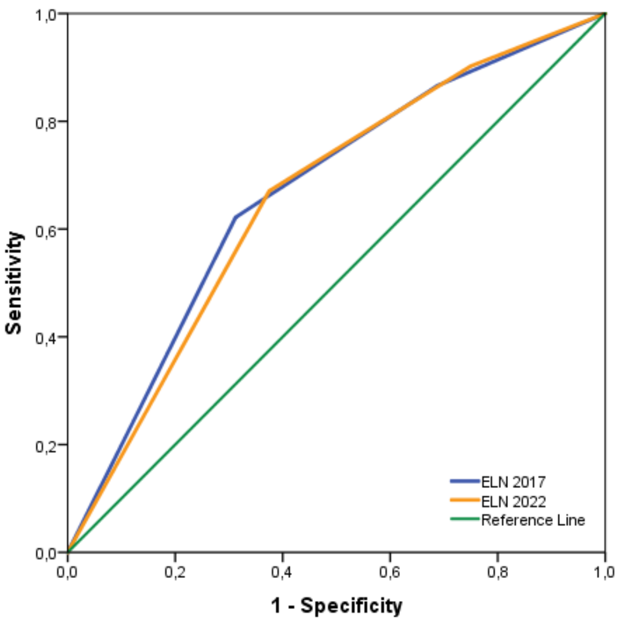

Figure S5. ROC curves for OS in global cohort by ELN2022 and ELN2017.

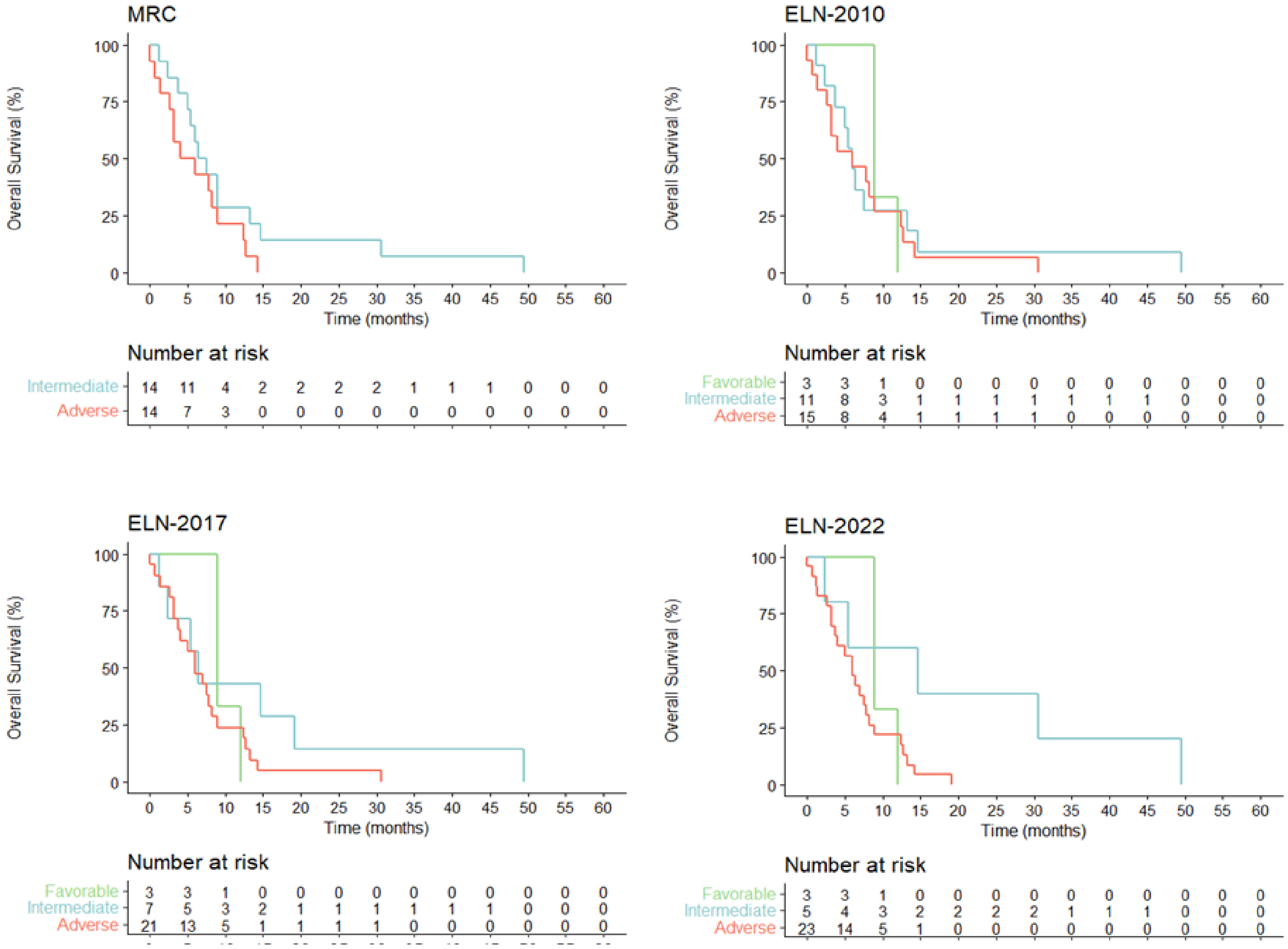

Figure S6. Overall Survival according to risk categories and classifications for patients receiving HSCT (*n* = 53).

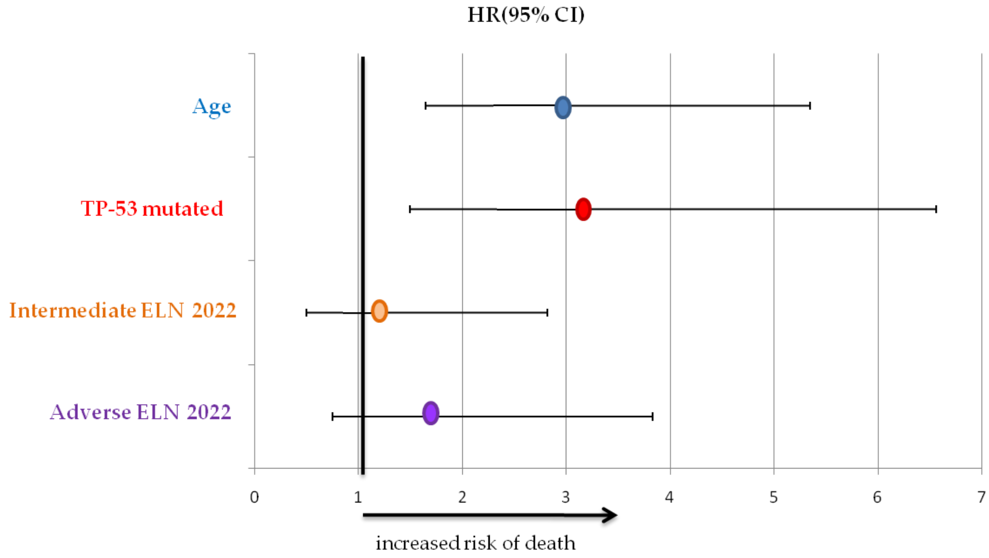

Figure S7. Overall Survival HRs obtained at multivariate analyses.

Table S1. Patient distribution of the entire cohort and according to ELN 2022 risk stratification with previous risk-classifications.

n (%)	ELN 2022 Favorable 20 (15.4)	ELN 2022 Intermediate 37 (28.5)	ELN 2022 Adverse 73 (56.1)	*p*
MRC cytogenetic classification				
MRC favorable	8 (40)	0	0	-
MRC intermediate	12 (60)	36 (100)	27 (39.7)	-
MRC adverse	0	0	41 (60.3)	-
Not classifiable				
ELN 2010 favorable	19 (95)	4 (10.8)	0	-
ELN 2010 intermediate I/II	1 (5)	31 (83.8)	31 (42.5)	-
ELN 2010 adverse	0	2 (5.4)	42 (57.5)	-
Not classifiable				
ELN 2017 favorable	20 (100)	6 (16.2)	0	-
ELN 2017 intermediate	0	25 (67.6)	13 (17.8)	-
ELN 2017 adverse	0	6 (16.2)	60 (82.2)	-

Table S2. Controversial mutational states according to ELN2010, ELN2017 and ELN2022 risk stratification.

n	%	ELN2010 Risk Stratification	ELN2017 Risk Stratification	ELN2022 Risk Stratification	Molecular Characteristics
1	0.07	Intermediate	Adverse	Adverse	Normal karyotype. Mutated *FLT3*-ITD$^{\text{high ratio}}$ + *SF3B1*
1	0.07	Intermediate	Adverse	Adverse	Normal karyotype. Mutated *FLT3*-ITD + *TP53*
1	0.07	Intermediate	Favorable	Intermediate	Normal karyotype. Mutated *NPM1*/*FLT3*-ITD$^{\text{high ratio}}$ + *SF3B1* + Biallelic mutated *CEBPA* no in frame bZIP
1	0.07	Adverse	Adverse	Adverse	Normal karyotype. Mutated *FLT3*-ITD$^{\text{high ratio}}$ + *RUNX1*
2	0.14	Favorable	Favorable	Favorable	Normal karyotype. Mutated *NPM1* + *SF3B1*
1	0.07	Favorable	Favorable	Favorable	Normal karyotype. *FLT3*-ITD$^{\text{low ratio}}$ + Biallelic mutated in frame bZIP *CEBPA*

Table S3. Overall Survival rate at 2 years and at 5 years and estimated mean and median of the intensively treated patients and detailed for each MRC cytogenetic, ELN2010, ELN 2017 and ELN 2022 risk group.

	OS % (SE)	Estimated Mean (CI 95%)	Estimated Median (CI 95%)
Global cohort	2y 53.6 (5.8)<br>5y 49.9 (6.3)	35.71 (29.6–41.72)	29.3 (NR)
MRC cytogenetic classification			
MRC favorable	2y 72.9 (16.5)<br>5y 72.9 (16.5)	42.74 (28.63–56.81)	Not reached
MRC intermediate	2y 60.5 (7.3)<br>5y5 0.4 (8.1)	38.99 (31.6–46.33)	Not reached
MRC adverse	2y 30.3 (10.4)<br>2y 30.3 (10.4)	23.09 (12–34.12)	10.6 (1.9–18.4)

		ELN2010 risk stratification	
ELN 2010 favorable	2y 73.7 (10.1)<br>5y 58.1 (12.7)	42.82 (31.36–54.28)	Not reached
ELN 2010 intermediate	2y 53.8 (8.9)<br>5y 47.8 (9.7)	37.37 (28.7–45.9)	43.5 (NR)
ELN 2010 adverse	2y 35.4 (10.5)<br>5y 35.4 (10.5)	25.96 (14.91–36.93)	11.6 (0.4–22.8)
		ELN2017 risk stratification	
ELN 2017 favorable	2y 66.0 (10.6)<br>5y 59.4 (11.4)	42.84 (32.1–53.56)	Not reached
ELN 2017 intermediate	2y 67.9 (9.6)<br>5y 50.9 (12.9)	38.98 (28.96–49)	Not reached
ELN 2017 adverse	2y 36.1 (8.8)<br>5y 36.1 (8.8)	27.82 (18.67–36.9)	18 (7.0–28.9)
		ELN2022 risk stratification	
ELN 2022 favorable	2y 75.1 (10.9)<br>5y 65.7 (12.9)	45.53 (33.24–57.83)	Not reached
ELN 2022 intermediate	2y 61.8 (10.3)<br>5y 48.5 (11.8)	40.46 (31.0–49.9)	43.5 (NR)
ELN 2022 adverse	2y 38.4 (8.3)<br>5y 38.4 (8.3)	27.5 (18.7–36.38)	13.6 (4.8–22.4)

SE, Standard error; CI, confidence interval; y, years.

Table S4. Overall Survival rate at 2 years and at 5 years and estimated mean and median of the intensively treated patients and detailed for each MRC cytogenetic, ELN2010, ELN 2017 and ELN 2022 risk group.

	OS %(SE)	Estimated Mean (CI 95%)	Estimated Median (CI 95%)
Global cohort	2y 53.6 (5.8)<br>5y 49.9 (6.3)	35.71 (29.6–41.72)	29.3 (NR)
		MRC cytogenetic classification	
MRC favorable	2y 72.9 (16.5)<br>5y 72.9 (16.5)	42.74 (28.63–56.81)	Not reached
MRC intermediate	2y 60.5 (7.3)<br>5y5 0.4 (8.1)	38.99 (31.6–46.33)	Not reached
MRC adverse	2y 30.3 (10.4)<br>2y 30.3 (10.4)	23.09 (12–34.12)	10.6 (1.9–18.4)
		ELN2010 risk stratification	
ELN 2010 favorable	2y 73.7 (10.1)<br>5y 58.1 (12.7)	42.82 (31.36–54.28)	Not reached
ELN 2010 intermediate	2y 53.8 (8.9)<br>5y 47.8 (9.7)	37.37 (28.7–45.9)	43.5 (NR)
ELN 2010 adverse	2y 35.4 (10.5)<br>5y 35.4 (10.5)	25.96 (14.91–36.93)	11.6 (0.4–22.8)
		ELN2017 risk stratification	
ELN 2017 favorable	2y 66.0 (10.6)<br>5y 59.4 (11.4)	42.84 (32.1–53.56)	Not reached
ELN 2017 intermediate	2y 67.9 (9.6)<br>5y 50.9 (12.9)	38.98 (28.96–49)	Not reached
ELN 2017 adverse	2y 36.1 (8.8)<br>5y 36.1 (8.8)	27.82 (18.67–36.9)	18 (7.0–28.9)
		ELN2022 risk stratification	
ELN 2022 favorable	2y 75.1 (10.9)<br>5y 65.7 (12.9)	45.53 (33.24–57.83)	Not reached
ELN 2022 intermediate	2y 61.8 (10.3)<br>5y 48.5 (11.8)	40.46 (31.0–49.9)	43.5 (NR)

ELN 2022 adverse	2y 38.4 (8.3)<br>5y 38.4 (8.3)	27.5 (18.7–36.38)	13.6 (4.8–22.4)

SE, Standard error; CI, confidence interval; y, years.
